# Mitochondrial genome and polymorphic microsatellite markers from the abyssal sponge *Plenaster craigi* Lim & Wiklund, 2017: tools for understanding the impact of deep-sea mining

**DOI:** 10.1007/s12526-017-0786-0

**Published:** 2017-09-30

**Authors:** Sergi Taboada, Nathan J. Kenny, Ana Riesgo, Helena Wiklund, Gordon L. J. Paterson, Thomas G. Dahlgren, Adrian G. Glover

**Affiliations:** 1 Life Sciences Department, The Natural History Museum, Cromwell Road, London, SW7 5BD UK; 2 Uni Research, PO Box 7810, 5020 Bergen, Norway; 3 Department of Marine Sciences, University of Gothenburg, Box 463, 40530 Gothenburg, Sweden

**Keywords:** Clarion-Clipperton Zone, Polymetallic nodules, Conservation genetics, Population genetics, Next-generation sequencing, Marine protected area

## Abstract

The abyssal demosponge *Plenaster craigi* is endemic to the Clarion - Clipperton Zone (CCZ) in the NE Pacific, a region with abundant seafloor polymetallic nodules and of potential interest for mining. *Plenaster craigi* encrusts on these nodules and is an abundant component of the ecosystem. To assess the impact of mining operations, it is crucial to understand the genetics of this species, because its genetic diversity and connectivity across the area may be representative of other nodule-encrusting invertebrate epifauna. Here we describe and characterize 14 polymorphic microsatellite markers from this keystone species using Illumina MiSeq, tested for 75 individuals from three different areas across the CCZ, including an Area of Particular Environmental Interest (APEI-6) and two areas within the adjacent UK1 mining exploration area. The number of alleles per locus ranged from 3 to 30 (13.33 average alleles for all loci across areas). Observed and expected heterozygosity ranged from 0.909–0.048 and from 0.954–0.255, respectively. Several loci displayed significant deviation from the Hardy-Weinberg equilibrium, which appears to be common in other sponge studies. The microsatellite loci described here will be used to assess the genetic structure and connectivity on populations of the sponge across the CCZ, which will be invaluable for monitoring the impact of mining operations on its habitat. Also, we provide the annotated mitochondrial genome of *P. craigi*, compare its arrangement with other closely related species, and discuss the phylogenetic framework for the sponge after Maximum Likelihood and Bayesian Inference analyses using nucleotide and amino acid sequences data sets separately.

**Electronic supplementary material:**

The online version of this article (10.1007/s12526-017-0786-0) contains supplementary material, which is available to authorized users.

## Introduction

The abyssal area between the Clarion - Clipperton Zones (CCZ) in the equatorial NE Pacific spans approximately 6 million km^2^, and encompasses a broad range of habitats including hills, seamounts, fracture zones, and extensive abyssal plains (Wedding et al. 2013). Abyssal plains deeper than 4000 m are covered by polymetallic nodules (potato-sized concretions of manganese, iron, nickel, copper and cobalt) and represent one of the most important areas for deep-sea mining exploration worldwide, with mining operations expected to start by 2025 (Borowski and Thiel 1998; Smith and Demopoulos 2003; Glover and Smith 2003; Smith et al. 2008).

Small-scale impact experiments conducted so far suggest that the direct environmental consequences of nodule mining will be severe and potentially long-lasting (Borowski and Thiel 1998; Thiel et al. 2001; Miljutin et al. 2011) and will directly affect abyssal epifauna (Vanreusel et al. 2016). Importantly, mining may impact not only areas of the seafloor owing to direct mining disturbance (at scales of 300–600 km^2^ per year), but will also disturb adjacent areas through re-deposition from sediment plumes 10–100 km from the mining site (Rolinski et al. 2001; Sharma et al. 2001). These concerns led to the suggestion and establishment of a network of deep-sea marine protected areas termed Areas of Particular Environmental Interest (APEIs) across the CCZ designed to safeguard the biodiversity and ecosystem functionality in this particular region (Wedding et al. 2013). In broad geographic areas such as the CCZ, the assessment of biogeographic patterns and larval dispersal of the different species is crucial to reduce impact on the biodiversity (Wedding et al. 2013). To achieve that, rigorous evaluation of species’ ranges and their levels of population connectivity and turnover is needed.

The recently described abyssal demosponge *Plenaster craigi* Lim & Wiklund, 2017 (Lim et al. 2017), belonging to the family Stelligeridae, represents an excellent model species for the assessment of molecular connectivity and the establishment of biogeographic patterns (spanning local through regional spatial scales) within the CCZ for a number of reasons. *Plenaster craigi* is a common encrusting element of the nodule fauna, probably endemic to this region, highly (perhaps totally) dependent on nodules, which provide the substrate for attachment of adults. Thus, after mining, populations of this organism will be eliminated from the mined areas as nodules are removed. The filter-feeding nutritional strategy of adults is also likely to make these organisms vulnerable to sediment re-deposition as the sediment plumes generated by mining may impact water flows and food particle filtering capacity (Bell et al. 2015; Pineda et al. 2016). Further, although nothing is yet known about its reproductive traits, as in other sponges *P. craigi* is assumed to have a dispersal phase through a lecithotrophic larva (Maldonado 2006), that might confer this species with limited dispersal ability.

Here we describe the isolation and characterization of a set of microsatellite loci using Illumina MiSeq high-throughput DNA sequencing for assessing genetic diversity and connectivity of the sponge populations, and test their performance in two distant areas within the CCZ (approx. 800 km), the APEI-6 and UK-1 exploration areas (Smith et al. 2013; Glover et al. 2015; Jones and Scientists 2015). In addition, we assembled and determined the complete mitochondrial genome of *P. craigi* to be screened for mitochondrial markers suitable for population genetic studies in the future, and also placed the sponge within its phylogenetic context using other already available sponge mitochondrial genomes. These resources will form the basis for more thorough investigation on the diversity, distribution and resilience of *P. craigi* to anthropogenic activity in its habitat.

## Material and methods

### Sample collection and preservation

The 75 specimens of the demosponge *Plenaster craigi* used in this study were collected from three different areas within the CCZ: APEI-6, UK1 Stratum A and UK1 Stratum B (Table 1). All specimens were found attached to polymetallic nodules that were mainly collected using an USNEL-type spade box core (0.25 m^2^). Nodules were observed with fauna maintained alive in cold-filtered seawater (Glover et al. 2015) under LED lighting and with the aid of macro-photographic cameras and stereo microscopes. When sponges were found they were photographed, removed from the nodule with a scalpel, preserved in 80% ethanol and RNALater, and immediately stored at −20 °C until DNA extraction.

**Table 1:** Loci characteristics and summary statistics of 14 primer pairs amplifying microsatellite loci in *Plenaster craigi*. *N* sample size, *Na* the number of alleles per locus, *He* expected heterozygosity, *Ho* observed heterozygosity, *FIS* inbreeding coefficient

Optimization details		Fluor.	Repeat Motif	Size Range	APEI-6				UK1 Stratum A				UK1 Stratum B			
Locus	F and R primers				N	Na	Ho/He	F_IS_	N	Na	Ho/He	F_IS_	N	Na	Ho/He	F_IS_
1Ple	ATATCTTGGTTCTGGCTGAGGA	6-FAM	(TCC)*9	169–232	25	9	0.640/0.808	0.208	22	9	0.909/0.782	−0.162	24	10	0.667/0.753	0.115
	GAGAAACCAGAGGACCAACAAC	–														
2Ple	GCACAATGTGGTGAGTCAGATT	6-FAM	(TACA)*16	176–236	25	8	0.440/0.680	0.353	25	6	**0.120/0.589**	0.796*	25	6	0.280/0.255	−0.097
	CCATTTGGACTTAGCATTTCAA	–														
3Ple	CATCTGCTTCTTCCCCTCATAC	6-FAM	(CA)*17	284–402	25	26	0.800/0.943	0.152	24	21	**0.625/0.907**	0.311*	25	21	**0.680/0.878**	0.226*
	TTCCCTCACCTTAATCCTCTCA	–														
4Ple	AAGTGCTCTGAGATTCCATGCT	6-FAM	(GAT)13	348–414	18	12	**0.278/0.872**	0.681*	23	14	**0.348/0.902**	0.614*	20	13	**0.300/0.879**	0.659*
	GTTGCAATGACCTACCTCGTTA	–														
5Ple	TGCACAGGCACTACTGAGGTAT	6-FAM	(AC)*12	449–485	25	4	0.240/0.423	0.433	24	3	**0.208/0.452**	0.539*	25	5	**0.360/0.523**	0.312*
	ACAGTGTGTTCCAGGCCTAGTT	–														
6Ple	ATGTTGCGAGTGATCTGTTGTT	6-FAM	(GT)*14	182–310	25	29	0.680/0.950	0.285	25	30	**0.840/0.946**	0.112*	25	24	**0.840/0.938**	0.105*
	GTCCAGCTGCTACAAGGGTTAC	–														
8Ple	ATTGCTTGCACACATTAACTGC	6-FAM	(CA)*16	308–484	24	24	**0.250/0.951**	0.737*	24	28	**0.583/0.954**	0.389*	25	23	**0.560/0.942**	0.405*
	GATGTTTTTCATCACCCAGGTC	–														
10Ple	CCTTCTCTCCACTCCTCTTTCA	6-FAM	(CCT)*13	403–469	25	11	**0.320/0.839**	0.619*	25	11	**0.640/0.854**	0.251*	25	9	0.640/0.782	0.182
	GTAGCTGTGTTGGTTTGGTGAG	–														
11Ple	TATGGGAGTTACGGAAGGAAAA	6-FAM	(TAT)*9	162–234	21	6	**0.190/0.757**	0.749*	22	7	0.409/0.754	0.458	25	8	**0.160/0.772**	0.793*
	GCCACAGAGTCAGACAATCAGA	–														
12Ple	AATGAGGATCTCCACTGCATCT	6-FAM	(GT)*26	213–337	25	18	0.800/0.898	0.110	22	16	**0.636/0.903**	0.295*	23	13	**0.478/0.833**	0.426*
	CTGCGAACTCCACTACACTACG	–														
13Ple	AACAGCCATGTGAGTTCAGCTA	6-FAM	(GAG)*9	323–371	25	8	**0.360/0.684**	0.474*	24	8	0.625/0.645	0.031	25	6	0.520/0.510	−0.020
	TGCTAGTGTTTCGAACAAGGAA	–														
14Ple	CATTTACGTAGCCCCAAGTCAT	6-FAM	(TGG)*11	428–461	25	7	0.640/0.698	0.084	25	6	**0.440/0.633**	0.305*	25	6	0.760/0.691	−0.100
	AAGGTGCTGTGCTGATGATCTA	–														
16Ple	ATAGCCAGGAAGTCCTTCAGC	6-FAM	(TG)*35	232–370	25	28	0.880/0.954	0.077	25	22	0.800/0.938	0.147	25	25	**0.800/0.930**	0.140*
	GACCATTAAACCAGGAGTGCAT	–														
19Ple	TGCAGCCCAGTAACATGTAAAA	6-FAM	(TCC)*10	281–311	24	7	**0.375/0.797**	0.529*	24	8	**0.083/0.785**	0.894*	21	5	**0.048/0.713**	0.933*
	AGTATTCGGTGGCATTTTCAAC	–														
Total					24.1	14.1	0.492/0.804	0.392*	23.9	13.5	0.519/0.789	0.356*	24.1	12.4	0.507/0.743	0.291*

*Significant deviation from Hardy-Weinberg Equilibrium after application of Narum correction (*P* < 0.05). Locus showing significant excess of homozygotes in bold

### DNA extraction and Illumina sequencing

Genomic DNA was extracted from a piece of a single individual (7.77 mm long) collected from UK1 Stratum A using the DNEasy Blood & Tissue Kit (QIAGEN, Venlo, Netherlands) following the protocol provided by the manufacturer. DNA concentration was quantified using the Quant-iT dsDNA HS Assay Kit and read in a Qubit 2.0 Fluorometer (Life Technologies, Carlsbad, California) following the manufacturer’s instructions. After purification, a total of 200 ng of DNA was used for library preparation. Library preparation was performed using the Illumina Truseq Nano library prep kit according to the manufacturers’ protocol, with TruSeq Index AD015 used to allow later demultiplexing. Library insert size was 913 bp. Approximately 30% of one lane of Illumina MiSeq using the 2 × 300 bp paired-end length sequencing configuration was used to sequence this sample.

The DNA used for primer performance testing of the microsatellite markers (see below) was extracted from a subsample of tissue from each of the 75 individuals studied here (25 individuals from each of the three different areas: APEI-6, UK1 Stratum A and UK1 Stratum B), using the DNEasy Blood & Tissue Kit as described above.

### Microsatellite discovery

The Illumina MiSeq run yielded 8.617.658 paired reads, with 95% of the reads with a Phred quality score > 35 (> 99.9% base call accuracy). Adapter trimming and quality filtering was done using Trimmomatic v. 0.32 (Bolger et al. 2014) with the following settings: ILLUMINACLIP:Adaptor.fa:2:30:10 LEADING:3 TRAILING:3 SLIDINGWINDOW:4:15 MINLEN:36. Reads were assembled de novo into contigs using IDBA-UD assembler (min *k*-mer = 60; max *k*-mer = 300) (Peng et al. 2012). The highest *k*-mer size (300 bp) yielded 103.025 contigs, which were filtered for microsatellite discovery, and motifs with a minimum of 8 repeats were found using Phobos v. 3.3.11 (http://www.rub.de/spezzoo/cm/cm_phobos.htm). A total of 21.148 contigs contained microsatellites (93.41% dinucleotide, 0.89% trinucleotide, 5.63% tetranucleotide, 0.02% pentanucleotide and 0.05% hexanucleotide) and from these 20 sets of microsatellite primers were designed using the software PRIMER 3 (Untergasser et al. 2012) for 6 di-, 11 tri- and 3 tetranucleotide loci.

### Primer testing

PCR amplification success for the 20 sets of microsatellite primers was tested for 75 individuals from the three different areas using the following conditions: 94 °C / 3 min, (94 °C / 30 s; 45–60 °C / 35 s; 72 °C / 30 s)* 32 cycles, 72 °C / 10 min. PCR reaction mix contained 8.4 μL of Red Taq DNA Polymerase 1.1× MasterMix (VWR), 0.3 μL (10 μM) of fluorochrome-labeled forward primer (6’FAM), 0.3 μL (10 μM) of reverse primer with universal tail and 0.5 μL of extracted DNA. Amplification products were analyzed on an Applied Biosystems 3130xl DNA analyzer at the Molecular Facilities of the Natural History Museum (NHM) using a GS-500 (Thermo Fisher, Waltham, MA, USA) size standard. Allele peaks were checked and edited using Geneious v. 8.1.7 (http://www.geneious.com, Kearse et al. 2012) before being placed into amplicon size “bins” and exported for analysis. After an initial screening, 6 loci were excluded either due to PCR failure or ambiguous profiles when genotyping. The resulting 14 microsatellite loci used in the data analysis are listed in Table 1.

### Data analysis

Number of alleles per loci and area investigated, observed heterozygosity (*Ho*), expected heterozygosity (*He*) and fixation index (*F*_*IS*_) were calculated with GenAlEx (http://biology.anu.edu.au/GenAlEx/Welcome.html). Linkage disequilibrium among loci was calculated with ARLEQUIN vs 3.5.1.2 (Excoffier and Lischer 2010). The exact test for departure from Hardy–Weinberg Equilibrium (HWE) was performed with GenAlEx after applying a false discovery rate (FDR) correction using the B-Y method (Benjamini and Yekutieli 2001) as suggested by Narum (2006).

### Mitochondrial genome assembly, annotation and gene order analysis

BLAST searches (TBLASTN, all default settings) conducted using standalone BLAST+ (Camacho et al. 2009) on a local server, using sequences of known homology from other demosponge species retrieved from the *nr* nucleotide collection of GenBank, recovered a single contig within the IDBA-UD assembly containing the full mitochondrial genome of *P. craigi,* with some repeated sequences at either end of the single contig*.* To ensure the veracity of this assembly, raw reads were independently subjected to a stringent read cleaning process using Trimmomatic v. 0.32 with the following settings: ILLUMINACLIP:Adaptor.fa:2:30:10 LEADING:3 TRAILING:3 SLIDINGWINDOW:4:20 MINLEN:30 (where the Adaptor.fa file contained the appropriate Truseq sequences used for indexing). Velvet v. 1.2.10 (Zerbino and Birney 2008) was then run with a *k*-mer size of 91 using these cleaned reads. BLAST searches recovered three contigs in this Velvet assembly that together recapitulated the contig observed in IDBA-UD assembly, with no changes in the nucleotide sequence. The short repetitive sequence observed at both ends of the contig recovered by IDBA-UD was spanned internally within one Velvet contig, allowing clear confirmation of the complete sequence identity.

The resulting complete mitochondrial sequence was then annotated using the MITOS webserver (Bernt et al. 2013b) with the Coelenterate NCBI code for translation. Annotations were manually curated, particularly as start and stop codons were often not identified automatically. The circular mitochondrial genome representation was performed using OrganellarGenomeDRAW (Lohse et al. 2013), including the relative GC content. CREx (Bernt et al. 2007) was used to find the most parsimonious explanation for the gene arrangement seen in *P. craigi,* with the common intervals parameter used for distance measurement and the gene order of *Topsentia ophiraphidites* (de Laubenfels, 1934)*, Ectyoplasia ferox* (Duchassaing & Michelotti, 1864), and *Geodia neptuni* (Sollas, 1886), specifically used for hypothesizing the rearrangements necessary for its present sequence.

### Phylogenetic analyses based on mitochondrial sequences

Nucleotide sequences from *rrnL*, *rrnS* and all 14 mitochondrial protein coding genes, and amino acid sequences from the latter dataset, were used for phylogenetic reconstruction of heteroscleromorph demosponge inter-relationships using maximum likelihood and Bayesian methods. Known mitochondrial sequences (Supp. File 1) from 21 species of heteroscleromorph sponges, along with three Verongimorpha and a single member of the Keratosa subclasses (see Supp. File 2 for accession numbers and source details), were downloaded from NCBI’s *nr* database. Gene by gene, these sequences were aligned using the MAFFT online server (Katoh et al. 2002), under the FFT-NS-i method. The resulting alignments were then fed individually into Gblocks (Castresana 2000) with all three relaxed parameters used, to exclude ambiguous and excessively variable regions of the alignment. The resulting final 16 (nucleotide) and 14 (amino acid) alignments were then concatenated using FASconCAT (Kück and Meusemann 2010) to form final alignments of 15,393 bp and 3905 residues in length. All sequences and alignments are available as Supp. File 1 to this manuscript online, with NCBI accession numbers for the mitochondrial genomes used in the analysis available in Supp. File 2.

jModelTest2 (Darriba et al. 2012) and ProtTest 3.2 (Darriba et al. 2011) were then run on nucleotide and amino acid alignments to estimate the best fitting models of nucleotide and amino acid substitution (GTR + I + G and JTT + I + G, respectively). Maximum Likelihood (ML) analyses were conducted in RAxML v. 8.2.3 (Stamatakis et al. 2008) under these models with 1000 bootstrap replicates under the rapid bootstrapping mode. Bayesian Inference (BI) was also used to analyze phylogenetic inter-relationships, with nucleotide data further analyzed with Phylobayes v4.1 (Lartillot et al. 2009), under the CAT-GTR model, and both amino acid and nucleotide data were analyzed with four discrete gamma categories, maximum discrepancy 0.1 and minimum effective size 100. readpb was used to discard 20% of sampled points as ‘burn-in’ and remaining samples were used to generate averages for display.

## Results and discussion

### Microsatellites

To our knowledge, this is the first time that microsatellite markers have been developed for any benthic species inhabiting the CCZ, an extensive area in the equatorial NE Pacific where a range of different habitats occur (Wedding et al. 2013). To date, microsatellites have been successfully used to describe molecular connectivity and phylogeographic patterns in deep-sea marine invertebrates associated with hydrothermal vents in the SW Pacific or the Mid-Atlantic Ridge (e.g. Thaler et al. 2011; Teixeira et al. 2012). More recently, these markers have been isolated and developed with Illumina MiSeq technology and used to study the population genetic structure of two species of *Paralicella* amphipods from hadal trenches in the Pacific Ocean (Ritchie et al. 2016a, b).

Due to the proven low variability of cytochrome *c* oxidase I (*COI*) across the majority of sponges studied so far (Erpenbeck et al. 2006), most studies aiming to establish the molecular connectivity among sponge populations have developed microsatellite loci (e.g. Dailianis et al. 2011; Pérez-Portela et al. 2015; Riesgo et al. 2016). With the development of polymorphic microsatellites for *P. craigi* we provide a powerful tool to detect genetic connectivity at different scales (e.g. within and between APEIs and mining exploration areas). The data are useful to identify populations that are isolated and potentially more vulnerable to mining disturbances or sufficiently diverse and well connected to maintain regional genetic diversity and/or to facilitate the recovery of mined sites (Boschen et al. 2016).

Out of the 20 microsatellite loci attempted here, a total of 14 loci were optimized for their use in future molecular connectivity studies (Table 1). These loci showed no evidence of linkage disequilibrium across all pairwise comparisons. The number of alleles (*Na*) per locus varied from 30 in 6Ple to 3 in 5Ple (both in UK1 Stratum A) with an average of 13.33 alleles for all loci across all areas. Observed heterozygosity (*Ho*) ranged from 0.909 in 1Ple for UK1 Stratum A to 0.048 in 19Ple for UK1 Stratum B (mean value from 0.492 to 0.519), while expected heterozygosity (*He*) ranged from 0.954 in 16Ple and 8Ple for APEI-6 and for UK1 Stratum A, respectively, to 0.255 in 2Ple for UK1 Stratum B (mean value from 0.743 to 0.804). Mean values of *He* were slightly greater than those reported for other shallow-water sponges (e.g. Pérez-Portela et al. 2015; Riesgo et al. 2016) and from the deep-sea amphipod *Paralicella tenuipes* Chevreux, 1908 (Ritchie et al. 2016b), and similar to those reported for the shallow-water Mediterranean sponge *Spongia officinalis* Linnaeus, 1759 (Dailianis et al. 2011) or for the deep water hydrothermal vent gastropod *Ifremeria nautilei* Bouchet & Warén, 1991 (Thaler et al. 2011). Several loci showed significant departures from the Hardy-Weinberg equilibrium after FDR corrections and also showed heterozygosity deficit (Table 1), something that appears a common trait in other sponge studies as well as on many other marine benthic invertebrates, as has recently being discussed by Riesgo et al. (2016). Briefly, reasons explaining these high levels of homozygosity may include a significant effect of null alleles, high levels of inbreeding, selection against heterozygotes, the Wahlund effect or a combination of all of these factors (Freeland et al. 2011).

### Mitochondrial genome annotation

**Fig. 1:**
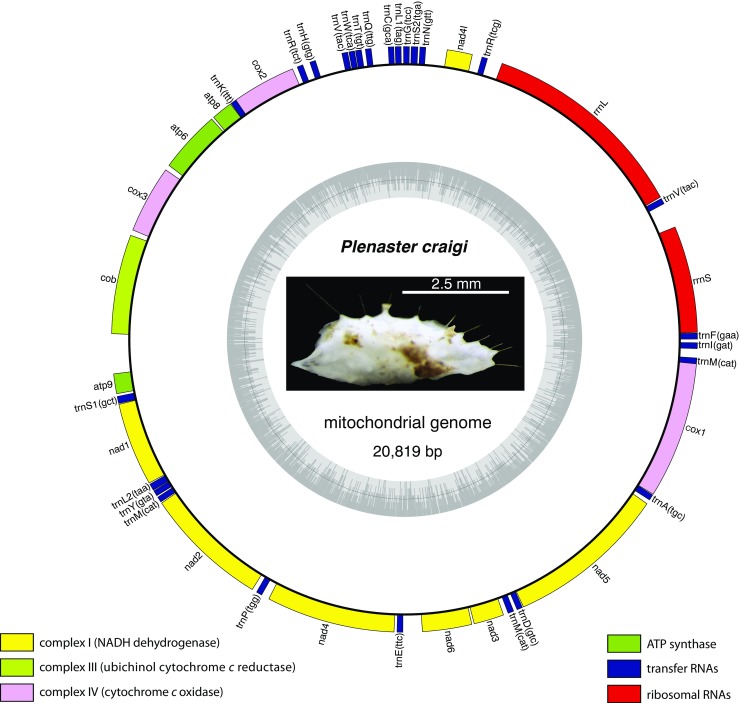
The *Plenaster craigi* circular mitochondrial genome with orientation of genes (all transcribed in same frame) represented by the outside circle. Local GC content (GC dark gray, AT light gray) represented on the inner ring. Gene families indicated by color legend at bottom. Photograph from a live specimen collected in the APEI-6 area

**Fig. 2:**
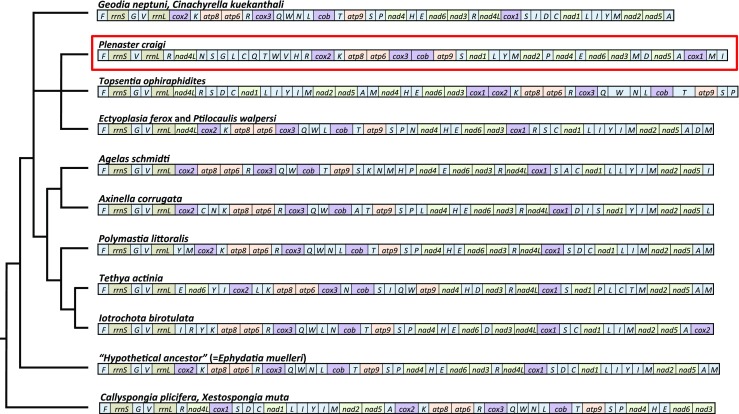
Heteroscleromorph demosponge, mitochondrial genome gene order, with that of *Plenaster craigi* boxed in red. Genes are color coded according to their families – tRNA genes in light blue, rRNA in light green, cytochrome oxidase and reductase genes in purple, NADH genes in green and ATP synthase genes in orange. A basic cladogram showing inter-relationships between these species is shown at left. For more detailed analysis see Fig. 3 and Supp. Fig. 1

The mitochondrial genome of *P. craigi* (Fig. 1) was 20,819 bp in length, slightly larger than that of most related species, but not among the largest examples of demosponge mitochondrial genomes, which can range up to almost 26 kilobases in length (e.g. see Lavrov et al. 2008). Its GC content was 7949 bp (38.18% of the total sequence), and a map showing the GC percentage around the entire molecule can be seen on the inner ring of Fig. 1. A total of 26 tRNA, 14 protein coding and 2 rRNA genes were present, and all genes possessed the same orientation. Of the protein coding genes, only one did not possess a standard ATG start codon (nad6, TTG), a trait shared with almost all other heteroscleromorph sponges. The total number of genes (42) observed was very high for a metazoan mitochondrial genome, due to tRNA gene duplication, although it is not unusual for a heteroscleromorph (Lavrov et al. 2008). The sequence of this mitochondrial genome has been uploaded to NCBI’s *nr* database under the accession number MF947452; the order of the genes around this molecule, along with gene start/stop sites are shown in Figs. 1, 2 and Supp. File 2 Table 1.

**Fig. 3:**
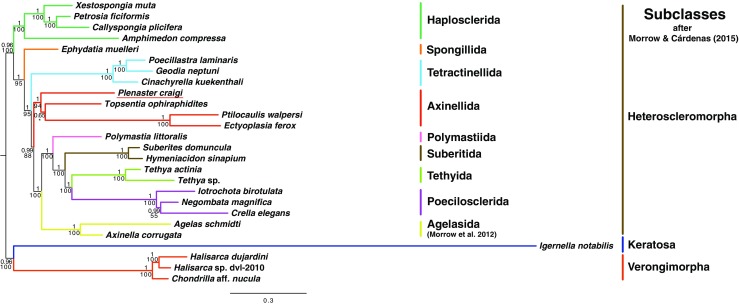
Phylogenetic inter-relationships between heteroscleromorph and outgroup demosponge species, inferred using both maximum likelihood (GTR + I + G, RAxML) and Bayesian (CAT-GTR, Phylobayes) analyses using a concatenated nucleotide alignment of all protein coding gene sequences along with those of *rrnL* and *rrnS*. Numbers at bases of nodes represent posterior probability (above) and bootstrap (below) support for nodes. Asterisk represents very poor (<10) bootstrap support, where a collapsed polytomy should be inferred as present in the ML tree. Names of orders and subclasses given at right, with *Plenaster craigi* underlined in red. Scale bar represents substitutions per site at given unit distance

The mitochondrial genomes of around 25 species of heteroscleromorph demosponges have been described previously (although Lubomirskiidae sponges from Lake Baikal described in Lavrov et al. 2012 were not included in our analysis), and generally exhibit a well-conserved gene order, the presence of *ATP9* and a shared coelenterate-like genetic code (Wang and Lavrov 2008, Zhang et al. 2016). The mitochondrial genome of *P. craigi* possessed many of these qualities and differed from previously described mitochondrial arrangements in gene order (e.g. Fig. 2, Wang and Lavrov 2008, Zhang et al. 2016). In particular, it differed in that a large number of tRNA genes were found in a ‘cluster’ rather than spread throughout the molecule. This ‘clustering’ can also be seen in *Topsentia ophiraphidites,* the closest species to *P. craigi* in our phylogenetic analysis (see Fig. 3 and discussion below), although the identity of these tRNA genes and the relative order of other genes does not seem to be conserved between these two species (Fig. 2). A number of tRNA genes, particularly methionine and valine, were present in higher numbers in *P. craigi* than in other sponge species. Arginine and serine were also duplicated, a trait that can be observed more widely in heteroscleromorphs (Lavrov et al. 2008). Despite overlap in the coding sequence between adjacent genes being commonly observed in heteroscleromorph genomes (e.g. four instances in *Negombata magnifica* (Keller, 1889); Belinky et al. 2008), only two instances of overlap were observed here, with *cox2*/*trnK* and *cox1*/*trnM*, both overlapping by 6 nucleotides.

Analyses of the possible evolutionary changes in pattern leading to the present arrangement of the mitochondrial genome of *P. craigi* were performed using the CREx tool (Bernt et al. 2007). Raw district matrices (including tRNA in the analysis) indicated that the arrangement of the mitochondrial genome of *P. craigi*, while differing markedly in gene order from that of other species, is more similar to that of other members of the Axinellida and early branching members of the heteroscleromorph clade (Fig. 2). For example, it is similar to *Topsentia ophiraphidites* (distance matrix score = 58), *Ectyoplasia ferox* and *Ptilocaulis walpersi* (Duchassaing & Michelotti, 1864) (56). It is also relatively similar to likely earlier-branching relatives such as *Geodia neptuni* (54), *Cinachyrella kuekenthali* (Uliczka, 1929) (54), *Ephydatia muelleri* (Lieberkühn, 1856) (54), *Callyspongia plicifera* (Lamarck, 1814) and *Xestospongia muta* (Schmidt, 1870) (56). By way of contrast, the highest scoring alternate ‘in group’ comparison, *Polymastia littoralis* Stephens, 1915*,* had a distance matrix score of 36. While further sampling of related species would allow firmer conclusions to be made about the evolution of the arrangement observed, CREx posits that three “tandem duplication-random loss” (tdrl) events would be sufficient to explain the diversification of its arrangement from that seen in *G. neptuni* and *C. kuekenthali,* and it is possible even fewer trdl events would be necessary to derive the current arrangement from that of the common ancestor of these species. The relative arrangement of the mitochondrial genomes of *T. ophiraphidites*, *E. ferox* and *P. walpersi* would all require three trdl events and a single transposition event, when compared to that of *P. craigi*. Therefore, despite the large variation in arrangement observed in these species, the number of changes required to explain the current gene orders is relatively small.

Group I and group II introns have already been reported in the mitochondrial genomes of some demosponges, including deep water species and members of the order Axinellida, in which *P. craigi* has been placed (see Schuster et al. 2015 and references herein). However, no evidence for the presence of similar introns was seen in our data. This reinforces the hypothesis of independent gain of these introns in the species in which they are found, likely by horizontal gene transfer (Erpenbeck et al. 2015), rather than ancestral presence across their lineages.

### Phylogenetic analyses

Using the sequences of the protein coding genes within this mitochondrial genome, along with the sequences of the rrnL and rrnS subunits, we undertook phylogenetic analysis of the inter-relationships between *P. craigi* and other heteroscleromorph sponges using both ML and BI methods. The results of these investigations can be seen in Fig. 3 (nucleotide sequences under the GTR + I + G/GTR-CAT models) and Supp. Figure 1 (amino acid sequences under the JTT + I + G model). These trees were essentially identical in basic topology, with the exception in the amino acid tree of a poorly resolved node at the base of the Tetractinellida (Supp. Fig. 1 shows a sister taxa relationship of Tetractinellida to the Axinellida, which is poorly supported –posterior probability 0.5 and bootstrap support <10–) and most likely an artifact, given the firm support values shown in Fig. 3.

The placement of *P. craigi* in the Axinellida seems to be firmly supported by our phylogenetic analyses, which corroborate the findings by Lim et al. (2017) who used an alignment of the 28S rRNA gene to estimate the phylogeny of *P. craigi*. Maximal posterior probability support and high bootstrap values (≤ 90) were found at the base of the Axinellida clade (Fig. 3). *Topsentia ophiraphidites* itself had previously been shown to be a member of Axinellida (Huchon et al. 2015) and with affinity to Desmanthidae (Schuster et al. 2015), unlike other *Topsentia* species, which were historically posited to be members of the Suberitida, although this placement may be in need of revision (Morrow and Cárdenas 2015). BI on both nucleotide and amino acid data supported *P. craigi* as the sister to a clade including *T. ophiraphidites*, *P. walpersi* and *E. ferox*, although ML analysis showed less support of this hypothesis with nucleotide data (Fig. 3, Supp. Fig. 1).

Inter-relationships within the Heteroscleromorpha are otherwise generally recovered as stated in Morrow and Cárdenas (2015). Our results further support Agelasida as proposed in Morrow et al. (2012), with *Axinella corrugata* (George & Wilson, 1919) strongly suggested as the sister species to *Agelas schmidti* Wilson, 1902 (Fig. 3, Supp. Fig. 1). We also found a well-supported structure to the relationships between a number of orders within Heteroscleromorpha (Haplosclerida, Poecilosclerida, Polymastiida, Suberitida, Tethyida, Tetractinellida and Agelasida; Fig. 3, Supp. Figure 1). However, it is worth mentioning that owing to present under-sampling of the full diversity of the clade, we did not include species from a number of orders and families within Heteroscleromorpha (see Morrow and Cárdenas 2015), all of which could give further information for the more complete understanding of heteroscleromorph phylogeny based on mitochondrial genome information.

## Conclusions

As deep-sea mining projects become a reality, we need new and robust methods to estimate their potential impact on the environment. Here we describe vital tools for the assessment of connectivity in the demosponge *P. craigi,* that will allow the inference of such impacts before, during and after mining operations. The 14 microsatellite loci described here have been tested and shown to be of utility for future molecular connectivity studies, which will allow the dispersal patterns to be mapped at both local and broader scales. Further, the description of the complete mitochondrial genome of this sponge will be useful for designing mitochondrial markers suitable for phylogenetic and phylogeographic studies and also for understanding how *P. craigi* has diverged from its closest relatives, as well as allowing us to more completely understand the phylogeny of the Demospongiae as a whole. Whether differences in the rearrangement of the mitochondrial genome are related to the phylogenetic position of *P. craigi* or to adaptations to life in its abyssal habitat should be investigated in the future by comparing the mitochondrial gene order of *P. craigi* with shallow water relatives. Using all the resources generated in our study, the regulating authorities of future mining operations can take into account local diversity when planning areas for exploitation, identify particularly vulnerable populations and avoid irreversible damage to the intriguing and unique ecosystem in which *P. craigi* is found.

## Electronic supplementary material

ESM 1 (PDF 190 kb)

ESM 2 (DOCX 81 kb)
