# Supplementary material for: Mitochondrial genome and polymorphic microsatellite markers from the abyssal sponge Plenaster craigi Lim & Wiklund, 2017: tools for understanding the impact of deep-sea mining
Source: Mar Biodivers. 2017 Sep 30;48(1):621–30. doi: 10.1007/s12526-017-0786-0 (PMC6445405; doi:10.1007/s12526-017-0786-0)
Supplement: Supplementary file 1 — (PDF 190 kb) [file 12526_2017_786_MOESM1_ESM.pdf]

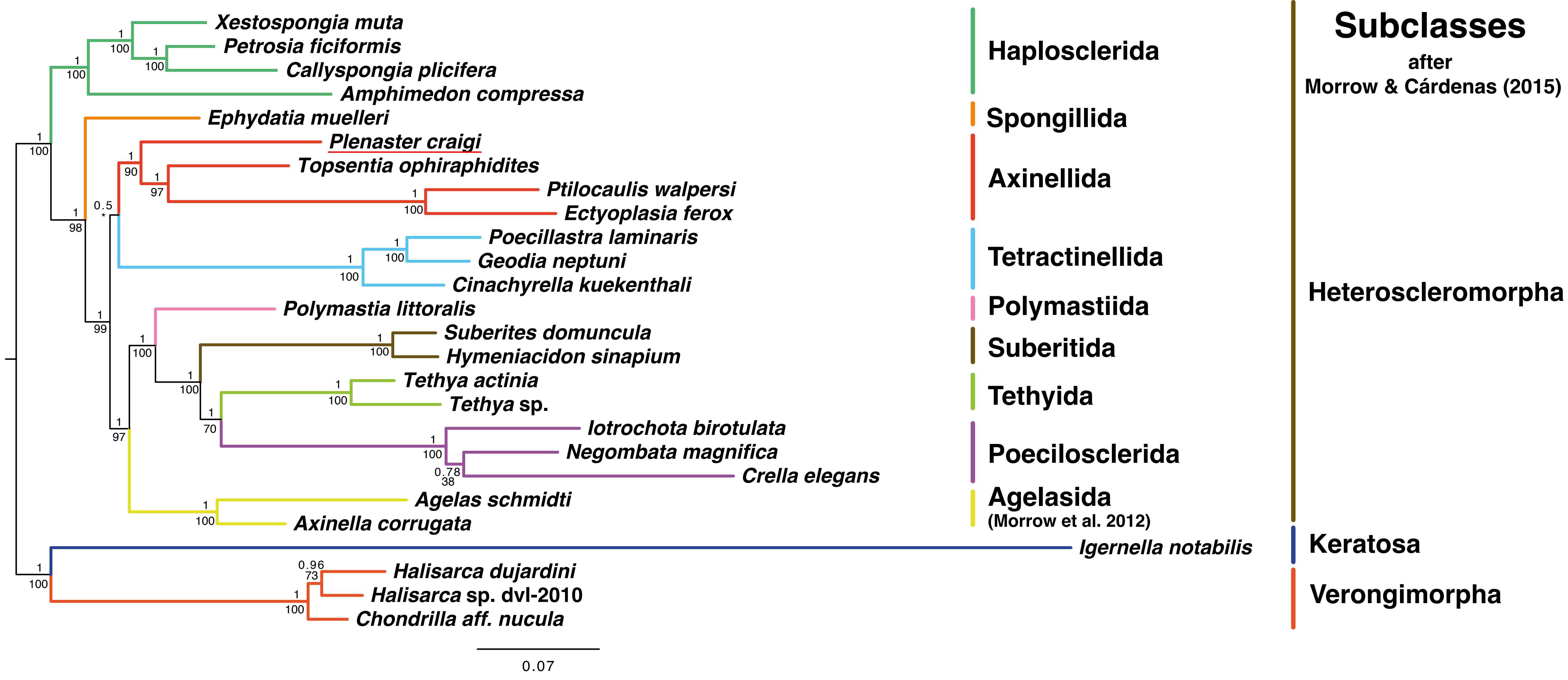
Xestospongia muta
Petrosia ficiformis
Callyspongia plicifera
Amphimedon compressa
Ephydatia muelleri
Plenaster craigi
Topsentia ophiraphidites
Ptilocaulis walpersi
Ectyoplasia ferox
Poecillastra laminaris
Geodia neptuni
Cinachyrella kuekenthali
Polymastia littoralis
Suberites domuncula
Hymeniacidon sinapium
Tethya actinia
Tethya sp.
Iotrochota birotulata
Negombata magnifica
Crella elegans
Agelas schmidti
Axinella corrugata
Igernella notabilis
Halisarca dujardini
Halisarca sp. dvl-2010
Chondrilla aff. nucula
0.07
Haplosclerida
Spongillida
Axinellida
Tetractinellida
Polymastiida
Suberitida
Tethyida
Poecilosclerida
Agelasida
(Morrow et al. 2012)
Subclasses
after
Morrow & Cárdenas (2015)
Heteroscleromorpha
Keratosa
Verongimorpha
