# Supplementary material for: Mitochondrial genome and polymorphic microsatellite markers from the abyssal sponge Plenaster craigi Lim & Wiklund, 2017: tools for understanding the impact of deep-sea mining
Source: Mar Biodivers. 2017 Sep 30;48(1):621–30. doi: 10.1007/s12526-017-0786-0 (PMC6445405; doi:10.1007/s12526-017-0786-0)
Supplement: Supplementary file 2 — (DOCX 81 kb) [file 12526_2017_786_MOESM2_ESM.docx]

**Supplementary Table 1.** Mitochondrial genome contents and relative arrangement

| Gene | Start | Stop | Strand | Length |
| --- | --- | --- | --- | --- |
| trnF(gaa) | 1 | 73 | + | 73 |
| rrnS | 76 | 1309 | + | 1234 |
| trnV(tac) | 1594 | 1666 | + | 73 |
| rrnL | 1678 | 4083 | + | 2406 |
| trnR(tcg) | 4250 | 4320 | + | 71 |
| nad4l | 4427 | 4723 | + | 297 |
| trnN(gtt) | 4961 | 5032 | + | 72 |
| trnS2(tga) | 5051 | 5131 | + | 81 |
| trnG(tcc) | 5147 | 5218 | + | 72 |
| trnL1(tag) | 5241 | 5313 | + | 73 |
| trnC(gca) | 5324 | 5392 | + | 69 |
| trnQ(ttg) | 5581 | 5652 | + | 72 |
| trnT(tgt) | 5693 | 5765 | + | 73 |
| trnW(tca) | 5771 | 5841 | + | 71 |
| trnV(tac) | 5850 | 5922 | + | 73 |
| trnH(gtg) | 6227 | 6298 | + | 72 |
| trnR(tct) | 6378 | 6450 | + | 73 |
| cox2 | 6501 | 7241 | + | 741 |
| trnK(ttt) | 7235 | 7307 | + | 73 |
| atp8 | 7309 | 7560 | + | 252 |
| atp6 | 7582 | 8304 | + | 732 |
| cox3 | 8359 | 9144 | + | 786 |
| cob | 9201 | 10349 | + | 1149 |
| atp9 | 10798 | 11031 | + | 234 |
| trnS1(gct) | 11057 | 11143 | + | 87 |
| nad1 | 11147 | 12112 | + | 966 |
| trnL2(taa) | 12121 | 12204 | + | 84 |
| trnY(gta) | 12208 | 12279 | + | 72 |
| trnM(cat) | 12292 | 12362 | + | 71 |
| nad2 | 12371 | 13807 | + | 1437 |
| trnP(tgg) | 13857 | 13929 | + | 73 |
| nad4 | 14015 | 15499 | + | 1485 |
| trnE(ttc) | 15527 | 15598 | + | 72 |
| nad6 | 15814 | 16386 | + | 573 |
| nad3 | 16407 | 16760 | + | 354 |
| trnM(cat) | 16810 | 16882 | + | 73 |
| trnD(gtc) | 16919 | 16989 | + | 71 |
| nad5 | 16996 | 18849 | + | 1854 |
| trnA(tgc) | 18893 | 18965 | + | 73 |
| cox1 | 18969 | 20546 | + | 1578 |
| trnM(cat) | 20540 | 20611 | + | 72 |
| trnI(gat) | 20712 | 20784 | + | 73 |

**Supplementary Table 2.** NCBI accession numbers and source of mitochondrial sequences

| **Species** | **Accession Number** | **Source** |
| --- | --- | --- |
| *Agelas schmidti* | EU237475 | Lavrov et al (2008) |
| *Amphimedon compressa* | EU237474 | Lavrov et al (2008) |
| *Axinella corrugata* | NC_006894 | Lavrov and Lang (2005) |
| *Callyspongia plicifera* | EU237477 | Lavrov et al (2008) |
| *Chondrilla aff. nucula* | EU237478 | Lavrov et al (2008) |
| *Cinachyrella kuekenthali* | EU237479 | Lavrov et al (2008) |
| *Crella elegans* | KR911862.1 | Pett and Lavrov (2015) |
| *Ectyoplasia ferox* | EU237480 | Lavrov et al (2008) |
| *Ephydatia muelleri* | EU237481 | Lavrov et al (2008) |
| *Geodia neptuni* | NC_006990 | Lavrov et al (2005) |
| *Halisarca* sp. dvl-2010 | HQ606142.1 | Ereskovsky et al (2011) |
| *Halisarca dujardini* | EU237483 | Lavrov et al (2008) |
| *Hymeniacidon sinapium* | KF192342.1 | Jun et al (2015) |
| *Igernella notabilis* | EU237485 | Lavrov et al (2008) |
| *Iotrochota birotulata* | EU237486 | Lavrov et al (2008) |
| *Negombata magnifica* | NC_010171.1 | Belinky et al (2008) |
| *Petrosia ficiformis* | KR911863.1 | Pett and Lavrov (2015) |
| *Poecillastra laminaris* | KM362735.1 | Zeng et al (2016) |
| *Polymastia littoralis* | KJ129611.1 | del Cerro et al (2016) |
| *Ptilocaulis walpersi* | EU237488 | Lavrov et al (2008) |
| *Suberites domuncula* | AM690374.1 | Lukic-Bilela et al (2008) |
| *Tethya actinia* | NC_006991 | Lavrov et al (2005) |
| *Tethya* sp. | KU748128 | Zhang et al (2016) |
| *Topsentia ophiraphidites* | EU237489 | Lavrov et al (2008) |
| *Xestospongia muta* | EU237490 | Lavrov et al (2008) |

*Additional citations:*

Belinky, F., Rot, C., Ilan, M. and Huchon, D., 2008. The complete mitochondrial genome of the demosponge *Negombata magnifica* (Poecilosclerida). *Molecular phylogenetics and evolution*, *47*(3), pp.1238-1243.

del Cerro, C., Peñalver, A., Cuevas, C., de la Calle, F., Galán, B. and García, J.L., 2016. Complete mitochondrial genome of *Polymastia littoralis* (Demospongiae, Polymastiidae). *Mitochondrial DNA Part A*, *27*(1), pp.312-313.

Ereskovsky, A.V., Lavrov, D.V., Boury-Esnault, N. and Vacelet, J., 2011. Molecular and morphological description of a new species of *Halisarca* (Demospongiae: Halisarcida) from Mediterranean Sea and a redescription of the type species *Halisarca dujardini*. *Zootaxa*, *2768*, pp.5-31.

Jun, J., Yu, J.N. and Choi, E.H., 2015. Complete mitochondrial genome of *Hymeniacidon sinapium* (Demospongiae, Halichondriidae). *Mitochondrial DNA*, *26*(2), pp.261-262.

Lavrov, D.V. and Lang, B.F., 2005. Transfer RNA gene recruitment in mitochondrial DNA. *Trends in Genetics*, *21*(3), pp.129-133.

Lavrov, D.V., Forget, L., Kelly, M. and Lang, B.F., 2005. Mitochondrial genomes of two demosponges provide insights into an early stage of animal evolution. *Molecular biology and evol*, *22*(5), 1231-1239.

Lukić-Bilela, L., Brandt, D., Pojskić, N., Wiens, M., Gamulin, V. and Müller, W.E., 2008. Mitochondrial genome of *Suberites domuncula*: palindromes and inverted repeats are abundant in non-coding regions. *Gene*, *412*(1), pp.1-11.

Pett, W. and Lavrov, D.V., 2015. Cytonuclear Interactions in the Evolution of Animal Mitochondrial tRNA Metabolism. *Genome biology and evolution*, *7*(8), pp.2089-2101.

Zeng, C., Thomas, L.J., Kelly, M. and Gardner, J.P., 2016. The complete mitochondrial genome of the deep-sea sponge *Poecillastra laminaris* (Astrophorida, Vulcanellidae). *Mitochondrial DNA Part A*, *27*(3), pp.1658-1659.
